# *Giardia* and *Cryptosporidium* in Neo-Tropical Rodents and Marsupials: Is There Any Zoonotic Potential?

**DOI:** 10.3390/life11030256

**Published:** 2021-03-20

**Authors:** Kegan Romelle Jones, Laura Tardieu

**Affiliations:** 1 Department of Food Production (DFP), Faculty of Food and Agriculture (FFA), St. Augustine Campus, University of the West Indies (UWI), St. Augustine, Trinidad and Tobago; l_tardieu@hotmail.com; 2 Department of Basic Veterinary Sciences (DBVS), Faculty of Medical Sciences (FMS), School of Veterinary Medicine (SVM), Mt. Hope Campus, University of the West Indies (UWI), Mount Hope, Trinidad and Tobago

**Keywords:** Didelphid, marsupial, hystricomorphic rodent, zoonotic, protozoan

## Abstract

Cryptosporidiosis and giardiasis have been identified as emerging diseases in both developed and developing countries. Wildlife has been highlighted to play a major role in the spread of these diseases to humans. This review aims to highlight the research findings that relate to *Cryptosporidium* spp. and *Giardia* spp., with a focus on (1) parasitism of neo-tropical hystricomorphic rodents and marsupials from the genus Didelphis and (2) prevention and treatment strategies for humans and animals for the neo-tropical region. It was found that there are few studies conducted on neo-tropical rodent and marsupial species, but studies that were found illustrated the potential role these animals may play as zoonotic carriers of these two parasites for the neo-tropical region. Thus, it is recommended that further studies be done to assess the threat of protozoan parasites in neo-tropical wildlife to humans and domestic animals, and to further determine the most effective prophylaxis adapted for the unique conditions of the region.

## 1. Introduction

It is estimated that of the emerging diseases affecting humans, 60% of these are zoonotic, with greater than 71% of these zoonotic diseases resulting from wildlife [1,2]. Two parasitic diseases with zoonotic potential are giardiasis and cryptosporidiosis. Both have been identified as gastrointestinal (GI) tract diseases that can be transmitted via water and through the fecal–oral route, and often have a wide range of wildlife that can serve as disease vectors or as reservoir hosts [3,4]. Cryptosporidiosis and giardiasis are not always symptomatic, but are often identified as asymptomatic or causing mild to moderate disease in their host species [5,6,7].

*Giardia* has a world-wide distribution, and is known for being one of the main causes of enteritis in both man and livestock [8]. A flagellated protozoan, it is usually transmitted via water that has been contaminated with cysts. *Giardia* cysts are highly contagious, spreading person to person and via contaminated food [9,10]. This parasite possesses two life stages; cyst and trophozoite [7]. There have been eight genetic groups (assemblages) of *Giardia duodenalis* (A–H) identified, with assemblages A and B considered to be zoonotic [11]. Symptoms of giardiasis include prolonged, chronic, watery diarrhea, nausea, vomiting, and pain in the upper abdominal area in immunocompetent individuals; however, in children with chronic infections, this disease can reduce quality of life with persistent growth retardation, cognitive impairment, and impaired immune responses [12,13,14]. Acute infections have been reported by Hanevik et al. [15], resulting in post-infectious syndromes, namely irritable bowel syndrome and chronic fatigue.

*Cryptosporidium* is an enteric protozoan parasite whose oocysts are often transmitted via water, with several species of *Cryptosporidium* spp. being identified in the water (reservoirs) in Brazil [16,17], and also in the water of swimming pools [18,19,20]. The parasite lifecycle and pathophysiology have been well described [21], and it has been found in over 150 vertebrate hosts [22]. *Cryptosporidium* oocysts have also been found to be resistant to most common disinfectants that are utilized for water treatment [22,23]. There have been at least 20 species and genotypes that have been reported in humans, including *C. meleagridis; C. felis; C. canis; C. cuniculus*; *C. ubiquitum*; *C. viatorum*; *C. muris*; *C. suis*; *C. fayeri*; *C. andersoni*; *C. bovis*; *C. scrofarum*; *C. tyzzeri*; *C. erinacei*; and *Cryptosporidium* horse, skunk, and chipmunk I genotypes [5,16,24,25]. The species identified above have been found in a wide range of hosts, from domestic to wild species. Some of these hosts include cats, dogs, guinea pig, rats, pigs, cows, horses, turkeys, skunks, and chipmunks. It must also be noted that in most cases, the animal host were reservoirs, with animals showing few clinical signs of infection. Two particular species have been reported as being responsible for most of the infections that have been found in humans and mammals: *C. parvum* and *C. hominis* [10,26]. Of these two, *C. parvum* has been identified as the zoonotic species, with *C. hominis* being the more anthroponotic species [25]. Infection with this parasite often leads to gastrointestinal symptoms in humans, and can even result in 40% mortality in livestock [27,28], with further economic loss in livestock due to reduced growth combined with treatment associated costs [29].

Along with *Giardia* spp., *Cryptosporidium* spp. appears to largely affect the young, the immunocompromised, and domestic animal species [30]. It has increasingly been associated with malnutrition in young patients, as malnourished children are often predisposed to infection and have a higher incidence of death [30]. Symptoms of cryptosporidiosis usually consist of diarrhea, abdominal cramps, nausea, vomiting, weight loss, and a low-grade fever [31]. In immunocompetent individuals, the disease usually lasts for 1–3 weeks; in immune deficient and malnourished children, however, the main symptom of diarrhea can be severe with a high chance of mortality [4,10]. To underline the importance and significant socioeconomic burden these parasites have in developing countries, the World Health Organizations Neglected Disease Initiative 2004 included them in its list of pathogens [32].

In the neo-tropical region, man is increasingly coming into contact with wildlife through hunting to satisfy the growing demand for protein in the form of “wild meat”, combined with the advance of agriculture and urbanization into more natural wild areas. As a result, wild animals are frequently being observed in human-occupied areas, and have developed synanthropic behaviors that increase the risk of transmission of infectious diseases and zoonotic pathogens to man and livestock. Yet little study has been conducted in these regions to determine the epidemiology of zoonotic parasites, which can greatly aide in developing preventative measures. The aim of this study was to examine the information that is available on the two gastrointestinal protozoan parasites *Cryptosporidium* and *Giardia* and their zoonotic potential in neo-tropical rodents like the capybara, agouti, and lappe, as well as marsupial species like *Didelphis* spp., with the further goal of identifying areas that require future study.

## 2. Occurrence of *Giardia* and *Cryptosporidium* in Selected Neo-Tropical Rodents

The capybara (*Hydrochoerus hydrochaeris*), lappe (*Agouti paca*/*Cuniculus paca*), and agouti (*Dasyprocta leporine*) have been identified as neo-tropical rodents with the potential to be domesticated [33]. Brown-Uddenberg [34] and Nogueira-Filho et al. [35] have shown that the agouti and the capybara can be reared intensively for their meat and hides. If these animals are to be farmed to produce meat for human consumption, then the parasites which have the zoonotic potential to affect man should be known. Parasites which can cause diarrhea in man include protozoan parasites, such as *Giardia* spp. and *Cryptosporidium* spp. Several endoparasites have been identified to inhabit the gastrointestinal tract of these neo-tropical rodents [36], but there are few reports on zoonotic protozoan parasites that affect the gastrointestinal tract of both man and these rodents.

The capybara is described as a semi-aquatic rodent, and many researchers have found both *Giardia* cysts and *Cryptosporidium* oocysts in their feces [37,38,39,40]. Rodriguez- Duran et al. [37] noted the prevalence of *Giardia* spp. (1.66%), while Meireles et al. [40] noted a prevalence of 5.52% for *Cryptosporidium* spp. The identification of *Cryptosporidium* spp. utilized molecular techniques, and it was identified as *C. parvum* subtype II, which is genetically similar to the bovine isolate [40]. The subtype identified in the capybara was considered to a zoonotic subtype [40]. Although *Giardia* spp. were identified using morphological characteristics, the species was unable to be classified using this technique [37].

Only a few studies have been conducted to identify the presence of these organisms. Da Silva et al. [41] identified *Giardia* spp. present in fecal samples of intensively reared agoutis. In the agouti, this parasite was determined by morphological analysis of cysts present in the feces. Using morphological techniques, the identity of the species remains unknown. Studies conducted in search of protozoan parasites examining the feces of the agouti failed to identify any *Giardia* cysts or *Cryptosporidium* oocysts [39,42]. It must be noted that the studies mentioned above had a small sample size, and the techniques used were based on morphological characteristics. There have been no reports that have found either *Giardia* spp. or *Cryptosporidium* spp. in the lappe, and little research has been done on these protozoan parasites in the lappe. The literature analyzed shows that there is little information on these parasites in neo-tropical rodents; where research has been done, identification has been through morphological techniques rather than molecular analysis.

*Cryptosporidium* has been identified as an emerging disease in both developed [43] and developing countries [44]. The species of *Cryptosporidium* that affects humans are *C. hominis* and *C. parvum* [43,44]. Wildlife has been highlighted to play a major role in the spread of this disease to humans [45,46]. Other species that have been identified in humans include *C. muris*, *C. andersoni*, *C. canis*, *C. meleagridis*, and *C. felis* [45].

These parasites have been identified in leafy vegetables, due to contamination of the soil [47], and also in the water of swimming pools [18]. The available detection methods are based on morphological and molecular analysis. However, due to the relatively small quantity of the oocysts shed in the feces, morphological identification is quite unreliable. Although molecular tools may be more accurate, they are often very costly. Contrastingly, cheaper methods of identification but are often more inaccurate and give false negative results.

## 3. Occurrence in *Didelphis* spp.

The opossum is a generalist species that can be found in a variety of different habitats in the neo-tropics [48]. The opossum is often described as a synanthrope, as it is frequently sighted near human dwellings in the region [1,49]. The omnivorous diet of this species is described as wide, diverse, and opportunistic, ranging from fruits to small animals to fecal matter [50,51]. 

Many studies have identified this species as a host or reservoir vector species for a plethora of endoparasites and infectious agents that can cause disease [1,49,52,53], including the two protozoan species *Cryptosporidium* and *Giardia*, which have both been reported in *Didelphis* opossums *D. albiventris* [7], *D. virginiana* [54], and *D. aurita* [1]. The opossums’ ability to act as a host for these two infectious parasites, combined with its varied diet and synanthropic behavior, make it a likely candidate for the spread of zoonotic parasites.

In general, *Cryptosporidium* studies in opossums are limited, with results varying depending on the species. Although not located in the neo-tropics, the more northerly opossum species *D. virginiana* has been positively identified as carrying a number of *Cryptosporidium* species and its genotypes [23]. Knox [23] further reported 44% infection with *Cryptosporidium* spp. amongst the samples collected in California. Contrastingly, studies conducted on the same species, *D. virginiana*, located closer to the neo-tropical realm in Mexico, were unable to identify *Cryptosporidium* or *Giardia* spp., although several other parasitic species were identified [49]. Differences in results may have been due to differing detection sensitivity methods, as well as the location the samples were obtained. Thus, Knox [23] studies collected samples from locations where water bodies or water were readily available, while Aragón Pech et al.’s [49] research examined species that were in areas that may have a been a bit drier, with fewer bodies of surface water available.

Dall’Olio and Franco’s [55] research in Brazil found that *D. aurita* and other marsupials were infected with *Cryptosporidium* oocysts; however, no specific details were given as to the specific parasite load or *Cryptosporidium* species that were found in *D. aurita*. Contrastingly, studies in Brazil by Lallo et al. [26] and Holsback et al. [56] were unable to detect any *Cryptosporidium* spp. in *D. aurita* or *D. marsupialis*, respectively. Both studies utilized co-proparasitological studies, and although Lallo et al. [26] were able to identify other parasitic microsporidia, they lacked a positive result for *Giardia* and *Cryptosporidium* spp. It could be proposed that this might have been due to the low sensitivity of the tools that were utilized for that study and low parasitic load of the specimens sampled.

The *Cryptosporidium* spp. *C. macropodum* and *C. fayeri* have been reported in Australian marsupials [57,58], but these are not considered in this paper to be species of great concern for the neo-tropics, as they have been reported to only infect marsupial hosts from the region of Australia, which is outside the scope of this review. With regard to *Giardia*, infectious parasite eggs have been reported in *D. aurita* [59]. However, earlier studies suggest that this parasite does not significantly infect the opossum species *D. albiventris* and *D. marsupialis*, as Sogayar and Yoshida [60] found no evidence in fecal and intestinal scraping specimens taken from two different regions in the southwestern region of Brazil. This research, however, does not go into much detail on the sensitivity of the testing that was utilized or on how the fecal and intestinal samples were obtained and stored. Thus, the sensitivity of the tests may have been a factor in this study, being unable to identify the presence of *Giardia* spp.

Co-parasitism or mixed infections by two or more protozoan parasites has been proposed by Zanette et al. [7] to favor infection by *Cryptosporidium* spp. in opossums. This is supported by their research in Brazil (Rio Grande do Sul state), where both cysts of *Giardia* and *Cryptosporidium* spp. were positively identified in the wild-caught opossum *D. albiventris*. Similarly, multiple parasites were also found in studies by Yai et al. [61], where 10.58% of *Didelphis* spp. Captured in urban areas were infected with *C. parvum* along with other parasites. Zanette et al. [7] studies also observed the presence of oocysts and cysts from both *Giardia* and *Cryptosporidium*, along with *Eimeria* spp. Moreover, Aragón Pech et al. [49] identified polyparasitism in *D. virginiana* opossums in Mexico, but the species did not belong to either *Giardia* or *Cryptosporidium* genus. The effects of multiple parasitism favoring infection from protozoan species like *Cryptosporidium* warrants further study.

Geographic location has been identified as a factor, with one paper by Jimenez et al. [53] comparing two sympatric species of opossum (*Philander opossum* and *Didelphis marsupialis*). These studies determined that sympatric species have similar parasitic species and communities in common versus those found in the same species from different localities. Several investigators have identified *Cryptosporidium* using morphological analytical techniques. According to Dall’Olio and Franco [55], however, more sensitive, diagnostic, molecular-based techniques may be required to truly identify the presence of certain *Cryptosporidium* oocysts.

Studies by Aragón Pech et al. [49] found that time of year was associated with higher prevalence of parasites. This study proposed that later in the year, when higher humidity prevailed due to the rainy season, the newly weaned litters of opossums would get infected on their perambulations and search for food, and thus display higher parasitism levels than at other times of the year [49]. This theory, however, may only be for the temperate regions as this study was conducted in Mexico on the Virginian opossum, and may not be valid for the neo-tropics, which experience high temperatures and humidity year-round.

Although identified as having infectious potential to humans and livestock, some researchers have suggested that most *Cryptosporidium* spp. may be host-adapted and thus unable to be a major zoonotic source. Further to this, not all genotypes within a cluster may be infectious to humans, as many genotypes may be parasite–host-specific, and thus unable to have great zoonotic potential [62]. This is supported by Zanette et al.’s [7] studies on *D. albiventris*, which found that four out of the six species sampled carried parasites and displayed mild infection with no clinical symptoms.

Further support for this was found for New World opossums in earlier studies conducted by Lindsay et al. [63]. This study involved infecting young (*D. virginiana*) opossums with *C. parvum*, which resulted in mild pathogenic reactions. This indicated that juvenile opossums may possess an immunity to *C. parvum* infection, and therefore might not be prone to natural infection by this species. Nonetheless, further study is required to confirm this assumption.

Although opossums have been found to mainly act as hosts to *Cryptosporidium* and *Giardia* spp. in the above studies, the combined stresses of habitat loss and increased hunting pressure placed on this species in the tropics may lead these parasites to becoming pathogenic and zoonotic. Moreover, with limited studies conducted on neo-tropical species, many of the clinical aspects of both giardiasis and cryptosporidiosis in wild opossums in these regions have not been identified.

In summary, the opossum in the neo-tropics can be found in human-occupied areas, and yet little is known about the endoparasite population, prevalence, and the zoonotic potential this species likely presents. Most studies on neo-tropical opossums have focused on the South American congeners of species of *Didelphis*, i.e., *D. aurita* and *D. albiventris*, and were conducted on the mainland of South America, with no research being done on the smaller island opossum populations of *D. marsupialis* that are found in the Caribbean. Further studies are therefore required to determine the role of opossums with respect to pathogenicity and the effects that multi-parasitism of GI parasites can have on these animals, as well as their role as a reservoir of zoonotic pathogens.

## 4. Treatment/Prophylaxis and Prevention Strategies

Transmission of both of these parasites can either occur via the fecal matter ingestion route, or more often the oral route via water, food, or fomites [20,64]. Given the species mode of transmission and the demographic of the human population that it affects, treatment and prevention strategies for both *Cryptosporidium* and *Giardia* have been identified and are listed below.

In the treatment of giardiasis, the issue of antimicrobial resistance has been identified and described for this species [10]. Studies suggest that in areas where this species is endemic, a build-up of immunity might be occurring, as the symptoms of infection are less severe [10,65]; however, much greater research is needed to support this theory.

### 4.1. Water Treatment

Given that both of these parasites are waterborne, conventional water treatment is usually offered as a means of prevention. It should be noted that most reported waterborne outbreaks involving *Cryptosporidium* spp. have been in developed countries [19]. It can be argued that this may be due to better reporting in these regions compared to developing regions, where outbreaks may occur, but are unlikely to be identified and therefore do not get reported.

The oocysts of *Cryptosporidium* spp. have been found to be quite resistant to conventional water treatment techniques (coagulation, sedimentation, filtration, and chlorine disinfection) [66]. According to Betancourt and Rose [66], many great advances have been made in water treatment that can both detect and remove protozoa contamination in water sources. They supported a multi-barrier approach for these two parasites, including a combination of physical methods (filtration) and chemical (disinfectants). Studies that have looked at coagulation pre-treatment did find in some success with *Cryptosporidium* spp. removal, but it needed to be enhanced and operated under optimal conditions [67]. Kelly [68] proffers the suggestion that widespread use of technologies, such as boiling water, which has been found to be effective on *Cryptosporidium* oocysts, and ultraviolet sterilization for use in domestic settings might be possible preventative measures. However, to support the use and even feasibility of these measures in neo-tropical regions, more extensive studies are required. In terms of swimming pools, which have also been implicated quite widely in the spread of *Cryptosporidium* spp. [18,19,20,69], safe personal hygiene practices have been proposed to reduce or prevent the spread of disease in humans.

For larger bodies of water, studies by Graczyk et al. [70] have found success treating wastewater using natural treatment methods, via constructed wetlands or waste stabilization ponds. Additionally, the use of membrane technologies and ultrafiltration has also shown great potential, with a high removal of protozoan cysts from wastewater [71].

With *Cryptosporidium* spp. being notably resistant to chlorine [71,72], other disinfectants have been studied, and ozone has been found to be effective at inactivating *Cryptosporidium* oocysts, with a treatment of 1 ppm of ozone (1mg/L) for 5 min resulting in 90% inactivation of oocysts [71,72]. Similarly, UV irradiation has also been effective for inactivating *Cryptosporidium* oocysts [73]. Morita et al.’s [73] study further found that inactivation was most successful with a UV dose of 1.0 mWs/cm^2^ at 20 °C.

Overall, the most effective physical removal or inactivation of these protozoan parasites from water-borne sources would appear to be via the use of both water filtration and disinfection, preferably with ozonation and UV irradiation included. According to Carmena [32], these procedures may only work best once they are conducted under optimal conditions.

Finally, some researchers suggest that given the role wildlife plays, a greater focus needs to be on detection (quantification and identification) of cryptosporidiosis in wildlife excretions and in water samples, and that this will ultimately aide with estimations of *Cryptosporidium* spp. infection rates in water catchment areas. Other wildlife management strategies including population control, revegetation, and landscaping have been suggested [5]. Water catchment protection practices, like restricting animal access via fencing for both wildlife and domestic species, can aid in preventing the spread of protozoan parasitic diseases [32].

### 4.2. Drug Treatments (Cryptosporidium)

Children and immunocompromised individuals have been identified as the most at-risk groups in terms of infection by these parasites. Once infected with cryptosporidiosis, patients are given replacement fluids and electrolytes to treat diarrhea, which is the main symptom. The use of anti-motility drugs are also quite important. Some studies may supplement this treatment with narcotic agents, which may be effective for immunocompromised patients with autoimmune deficiency syndrome (AIDS). Proper nutrition is also strongly recommended for the treatment to be successful [30]. Some studies identify the use of a combination antiretroviral therapy as successful in removing parasites and reducing mortality in AIDS patients, but this is based on the assumption that restoring immune function will be vital to the management of cryptosporidiosis [30].

Research into anti-parasitic treatments is an area that is lacking in the region. The only drug that has been recognized for treatment of cryptosporidiosis is nitazoxanide, which is a broad-spectrum, anti-parasitic drug that through clinical trials has been found to be successful in clearing parasites from infected individuals [74]. Support for this is found in Rossignal’s [75] clinical trials, which demonstrate that nitazoxanide was effective at treating not only the main symptom of cryptosporidiosis, which is diarrhea, but also in reducing oocyst excretion in an immunocompetent cohort. However, this drug has been found to not be as successful in AIDS patients [76], as it requires an appropriate immune response. Given that anti-parasitic drugs have thus far had limited efficacy in immunocompromised patients infected with *Cryptosporidium* spp., much greater study is needed in this area.

Combinations of drug therapies have also had some marked success with treating young patients infected with *Cryptosporidium* spp., including azithromycin and nitrazoxanide or azithromycin and paromomycin [77,78]. Gargal [79] found combination therapy to restore immunity combined with antimicrobial treatment to be the best treatment for AIDS patients.

*Cryptosporidium* vaccines have been proposed, but appear to be hampered by the incomplete understanding of the host immune response to the parasite [5], and also by the wide variation in species that can lead to possible cross-reactions. Thus, further studies will be needed for an effective vaccine to be developed for cryptosporidiosis. With reference to prophylaxis for wildlife, no studies were identified, and this remains an area in need of research, particularly given that wildlife have been found to play a role in the lifecycle of *Cryptosporidium* parasites.

### 4.3. Drug Treatments (Giardia)

The most common antimicrobial drug treatment of giardiasis is derived from the 5-nitroimidazole (5-NI) family, which includes the drugs metronidazole and tinidazole, but reports of resistance to this group of drugs have been recorded, with up to 20% clinical resistance found [80,81,82]. Like *Cryptosporidium*, nitazoxanide has been utilized and has been found to be effective in clinical trials at reducing symptom duration for children displaying diarrheal illness [83]; however, other studies have cited its reduced efficacy, with 70–80% success [10,84]. Benzimidazoles, including albendazole and mebendazole, have been used to treat giardiasis with varying degrees of success (25–90%), dependent on the dosing regimen. Blackwell et al. [85] indicated that *Giardia* spp. increased when the treatment of mebendazole was administered for individuals infected with hookworms and *Giardia* oocysts. This was proposed by Blackwell et al. [85] to likely be evidence that there is an antagonistic relationship between helminths and *Giardia* spp., but this proposal requires greater study.

Quinacrine has been found to have some efficacy with treating giardiasis, with reports of 90% efficiency [10]. However, quinacrine may not be suitable for widespread use, as potentially severe adverse effects have been documented with its use. Consequently, quinacrine use has been effectively stopped in North America, with it no longer being commercially available [10]. It would appear that combination therapy might be the most effective treatment for this species, as this type of treatment appears to largely decrease the risk of developing antimicrobial drug resistance, but much greater research is required to confirm this assumption.

A *Giardia* vaccine for humans is not yet available, although a veterinary vaccine (GiardiaVax) is utilized for domestic species (dogs) and has been found to be effective at reducing both the symptoms and duration of cysts output. Miyamoto and Eckman [10] suggest that a veterinary vaccine might be effective for use post exposure. Overall, in terms of drug treatment and drug development, the goals are slightly different for these two protozoan parasites. For *Giardia,* although several classes of drugs exist that have been found to be effective, the dosing regimen and combination therapies need to be optimized. Meanwhile, with *Cryptosporidium*, one drug has been found to be effective, so more research needs to be conducted to identify other alternative and efficient drug treatments.

### 4.4. Probiotics

In terms of natural alternative treatments, the use of probiotics has been found to have some merit. A case study conducted by Pickerd and Tuthill [86] was able to successfully treat an immunocompetent patient diagnosed with cryptosporidiosis with a treatment of probiotics, resulting in a resolution of the infection. Although showing promise much greater study, using a wider cohort and a larger sample size is required for more conclusive support of the use of probiotics in the treatment of cryptosporidiosis.

Probiotic use for the treatment of Giardiasis has also been found in clinical animal model studies to have some success, with infected animals showing great promise [87]. Experimental studies by Goyal and Shukla [88] have found that oral administration of probiotics to *Giardia*-infected mice results in an anti-*Giardia* effect and an improved immune response, while Shukla and Shukla [88] studies found that a combination treatment of probiotics and antiprotozoal drugs resulted in enhanced recovery in mice models. Similar to cryptosporidiosis, more research is required to determine the exact mechanism by which probiotics modulate *Giardia* infection, to further determine the best probiotic or probiotic association and research using human clinical trials.

### 4.5. General Preventative Methods

Disinfection and cleaning are advised, and exposure to heat (45 °C) for 10–20 min have been found to completely inactivate oocysts [89]. *Cryptosporidium* oocysts are resistant to many disinfectants; however, disinfectants containing hydrogen peroxide and formaldehyde have been noted to have some inhibitory effect. Shahiduzzaman and Daugschies [28] cites Campbell et al. [90], which found ammonia and formalin to also reduce the infectious ability of oocysts. Shahiduzzaman and Daughschies [28] advise the best strategies for *Cryptosporidium* must be manifold, with therapy of exposed individuals to reduce spread, proper hygienic management, and measures to reduce the oocysts in the environment.

## 5. Conclusions

Cryptosporidiosis and giardiasis are important zoonotic diseases of both the developed and developing world. With human encroachment on wildlife habitats, as well as the captive rearing of wildlife species, there is an increased risk of zoonotic transmission of these microbes. As such, the identity of these species, along with their prevalence, must be known in neo-tropical species. Some research has been done with the capybara and the agouti. They have identified *C. parvum* type II and *Giardia* spp. in the capybara and *Giardia* spp. in the agouti. However, there is no literature that records these protozoan parasites in the lappe, which is an area that needs to be addressed. Further to this, molecular work needs to be done to identify the specific species and genotypes of *Giardia* and *Cryptosporidium*, which are present in the wildlife of the neo-tropics. It would appear that a better understanding of the environmental, epidemiological, and etiological factors that are associated with both *Giardia* and *Cryptosporidium* spp. are needed to best combat these parasites, and aid with developing more effective treatment and prevention methods.
